# The supraseptal ethmoid sinus cell: A previously unreported ethmoid sinus variant

**DOI:** 10.1002/ccr3.2215

**Published:** 2019-05-20

**Authors:** Mitchell R. Gore

**Affiliations:** ^1^ Department of Otolaryngology State University of New York‐Upstate Medical University Syracuse New York

**Keywords:** endoscopic sinus surgery, ethmoid sinus, migrated ethmoid sinus cell, variant ethmoid sinus cell

## Abstract

To the author's knowledge, this is the first reported case in the literature of a supraseptal ethmoid sinus cell, located between the nasal septum and ethmoid roof. Such a cell, while rare, should be recognized and dissected carefully to prevent skull base injury and cerebrospinal fluid leak.

## 1 INTRODUCTION

Variant ethmoid sinus cells can significantly affect the anatomy of the paranasal sinuses. Variant cells such as Onodi, Haller, and Kuhn cells can present a challenge when performing sinus surgery. Herein, we present a previously undescribed ethmoid cell lying superior to the nasal septum and inferior to the ethmoid roof.

To the author's knowledge, this is the first reported case of an ethmoid sinus cell superior to the nasal septum and inferior to the ethmoid roof/ethmoid skull base. The previously described ethmoid sinus cells, including Onodi cells (posterior ethmoid cells pneumatizing the superior medial or more commonly superior lateral sphenoid sinus),^1^, ^2^, ^3^ Haller cells (anterior ethmoid cells pneumatizing into the maxillary sinus),^4^, ^5^, ^6^ or Kuhn/frontal cells and agger nasi cells (anterior ethmoid cells pneumatizing into the frontal recess or frontal sinus)^7^, ^8^, ^9^ each present their own unique challenges when performing endoscopic sinus surgery, and recognition of the presence of these variant cells is important to complete sinus dissection and the avoidance of intraoperative complications. A search of the PubMed and Google Scholar databases with the keywords “supra‐septal cell” or “supra‐septal ethmoid cell” did not reveal any previous case reports of the supraseptal ethmoid cell.

Herein, we report a male patient with chronic rhinosinusitis and a left supraseptal ethmoid sinus cell who underwent functional endoscopic sinus surgery. Preoperative imaging demonstrated the cell and intraoperative navigation combined with careful endoscopic dissection facilitated thorough dissection of the cell without injury to the overlying ethmoid skull base.

## 2 CASE HISTORY/ EXAMINATION

**Figure 1:**
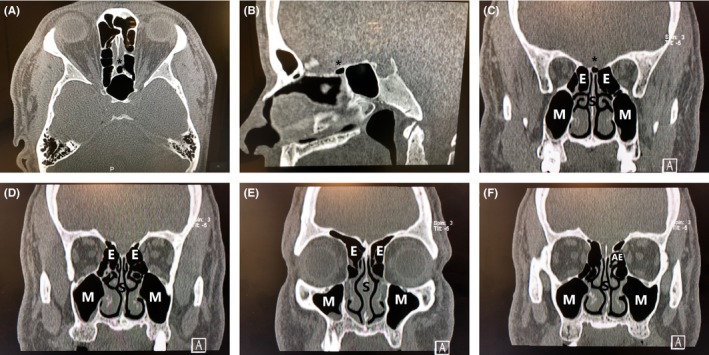
A, Axial computed tomography image of the left supraseptal ethmoid cell (labeled with star/*adjacent to the cell). B, Sagittal computed tomography image of the left supraseptal ethmoid cell (labeled with star/*superior to the cell). C, Coronal computed tomography image of the left supraseptal ethmoid cell (labeled with star/*superior to the cell). D, Coronal computed tomography image showing mucosal thickening/chronic rhinosinusitis in the right maxillary and ethmoid sinuses. E, Coronal computed tomography image showing mucosal thickening/chronic rhinosinusitis in the right greater than left maxillary and ethmoid sinuses. F, Coronal computed tomography image showing the left anterior ethmoid artery (AE) in the left ethmoid sinuses. M, maxillary sinus; E, ethmoid sinus; S, septum

A Hispanic male patient in his mid‐50s presented with chronic rhinosinusitis symptoms including postnasal drainage, facial pressure, and nasal congestion. He was treated with maximal medical therapy including 21 days of oral antibiotic therapy and intranasal steroids and had persistent symptoms at a follow‐up visit. Post‐treatment imaging demonstrated mucosal thickening in the maxillary and ethmoid sinuses consistent with chronic rhinosinusitis. The computed tomography (CT) of the sinuses also demonstrated an unusual supraseptal ethmoid sinus cell pneumatizing into the area between the superior aspect of the midnasal septum and the ethmoid roof. Figure 1A illustrates an axial CT view of the left supraseptal ethmoid cell. Figure 1B illustrates a sagittal CT view of the left supraseptal ethmoid cell. Figure 1C shows a coronal view of the supraseptal ethmoid cell with right greater than left maxillary and ethmoid mucosal thickening indicative of chronic rhinosinusitis. Figure 1D,E illustrates more anterior coronal CT slices demonstrating mucosal thickening indicative of chronic maxillary and ethmoid rhinosinusitis. Figure 1F demonstrates a coronal CT image showing the left anterior ethmoid artery running horizontally across the superior aspect of the left anterior ethmoid sinuses anterior and lateral to the left supraseptal cell. The patient was counseled on the risks, benefits, and alternatives of functional endoscopic sinus surgery and elected to proceed with surgery.

**Figure 2:**
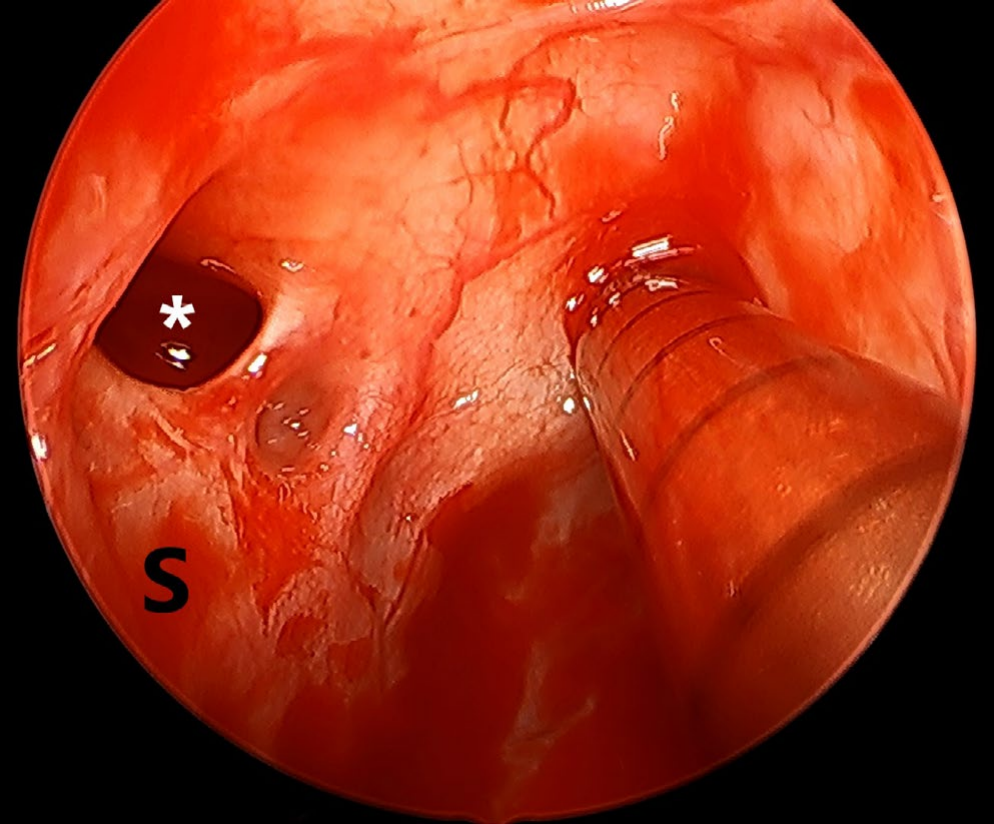
Intraoperative zero degree endoscopic view of the left supraseptal ethmoid cell (star/*). S, septum

During the endoscopic sinus surgery, the left supraseptal ethmoid sinus cell was identified (Figure 2). The cell was completely superior to the nasal septum, which typically attaches directly to the ethmoid roof. The bony prominence of the left anterior ethmoid artery was located superior and lateral to the supraseptal ethmoid cell in the fovea ethmoidalis/ethmoid roof (Figure 1F). Interestingly, close inspection of the supraseptal cell demonstrated a small additional partially pneumatized supraseptal ethmoid cell just lateral and posterior to the main supraseptal ethmoid cell, suggesting multiple cells in the area similar to the type II frontoethmoid/Kuhn cell.^8^, ^9^ The surgery was completed uneventfully, with thorough dissection of the bilateral maxillary, ethmoid, sphenoid, and frontal sinuses. The patient did well postoperatively with improvement in his nasal obstruction, nasal drainage, and facial pressure symptoms.

## 3 DISCUSSION

A thorough knowledge of paranasal sinus anatomy and associated anatomical variations is necessary to avoid incomplete dissection and complications when operating on the paranasal sinuses via the endoscopic approach. Familiarity with the ethmoid air cells and their variations helps prevent injury to nearby structures such as the anterior and posterior ethmoid arteries, the cribriform plate and fovea ethmoidalis, the orbital floor, and the optic nerves.

The ethmoid bone develops from the nasal capsule via ossification of the perpendicular plate of the ethmoid and the bilateral ethmoid labyrinths.^10^, ^11^ The labyrinths gradually form during fetal development and extend into the nasal conchae during the fourth and fifth months of fetal development. At birth, the ethmoid labyrinth is poorly developed, with the perpendicular plate of the ethmoid and crista galli beginning to ossify. The ethmoid labyrinth gradually pneumatizes during the pre‐adolescent years and is typically well pneumatized by the onset of puberty. We postulate that the supraseptal ethmoid cell is formed by hyper‐pneumatization of the ethmoid labyrinth into the perpendicular plate of the ethmoid during early ethmoid bone/ethmoid labyrinth development.

As with other well‐described variant ethmoid cells, the supra‐ethmoid sinus cell is clinically significant as its superior boundary is the central ethmoid skull base. Its unusual location makes recognition of this cell important, as dissection through the superior aspect of the supraseptal ethmoid cell may result in leakage of cerebrospinal fluid through the central ethmoid roof. Adequate dissection of the supraseptal ethmoid cell, if diseased or opacified, prevents persistent opacification of the cell or obstruction of posterior ethmoid cells by the supraseptal ethmoid cell.

To our knowledge, this specific ethmoid variant cell has not been previously described in the literature. In reviews of hundreds of preoperative maxillofacial CT scans, this patient had the single example of this particular ethmoid sinus cell noted by this author. The location of this particular ethmoid cell variant inferior and medial to the anterior ethmoid artery should be noted to avoid injury to the anterior ethmoid artery as it crosses the ethmoid roof (Figure 1F). The cell should be entered laterally to avoid injury to the cribriform plate or ethmoid roof. Preoperative imaging of the sinuses should be studied thoroughly in all three planes to familiarize the surgeon with the three‐dimensional sinus anatomy and any variant ethmoid sinus cells that may be encountered intraoperatively. If intraoperative navigation is utilized, this may facilitate intraoperative localization of these variant cells and aid thorough dissection of these ethmoid sinus cells.

## CONFLICT OF INTEREST

None declared.

## AUTHOR CONTRIBUTIONS

MRG: fulfilled the criteria and should qualify for authorship. MRG: conception and design, data acquisition, drafting the manuscript, and approving the revised and final version of the manuscript. The author has managed the manuscript submission process.

## ETHICS

This case report was determined to be exempt by the SUNY Upstate Institutional Review Board. The patient gave informed written consent to publish de‐identified information and clinical and radiographic images.
